# Middle School Academic Outcomes Related to Timing of English Language Acquisition in Dual Language Learners

**DOI:** 10.3390/bs15121612

**Published:** 2025-11-23

**Authors:** Gabriele Norvell, Tevis L. Tucker, Adam Winsler

**Affiliations:** Department of Psychology, George Mason University, Fairfax, VA 22030, USA

**Keywords:** dual language learners, DLL, ELL, academic performance, middle school, English proficiency

## Abstract

Dual Language Learners (DLLs) who become English proficient earlier experience better academic outcomes, but longitudinal research on the relationship between the timing of DLLs acquiring English proficiency and later academic outcomes while accounting for relevant factors is rare. We examined how the year in school in which DLL students (*N* = 14,852; 47% female; 85% in poverty; 88% Latinx, 8% Black, and 3% White/Asian/Other) acquired English proficiency (according to school system criteria) correlates with their later middle school (sixth–eighth grade) academic outcomes (GPA, standardized test scores, and grade retention), controlling for relevant factors. Earlier acquisition of English predicted better middle school outcomes and a lower likelihood of being retained in middle school. Some relations between the timing of English acquisition and outcomes were stronger for students not experiencing poverty. Implications for the education of DLL students in the U.S. and future research are discussed.

## 1. Introduction

Dual Language Learners (DLLs) in the United States are children who speak at least one language other than English at home and who are simultaneously mastering their native language(s) and gaining English (L2) proficiency (National Conference of State Legislatures [NCSL], 2018). Such children are often designated “English learners” (ELs) by school systems, but we will use the term DLL here to highlight the fact that there is more than one language involved. Estimates of the number of DLLs in the U.S. depend on whether the numbers include *former* DLLs—those who speak another language at home but who have reached the required proficiency level to no longer receive English as a second or other language (ESOL) services at school. There are over 5 million DLL students receiving ESOL services in schools across the United States, comprising about 11% of all students (National Center for Education Statistics [NCES], 2023). However, DLLs make up 32% of the population of students aged five–eight, including students already proficient in English (Migration Policy Institute, 2025). Identifying and meeting DLLs’ needs has been a significant concern within the last few decades, and understanding trajectories for DLLs’ language growth and academic achievement is an important first step. This present study investigated the extent to which the timing of English language acquisition is related to later academic achievement in middle school for DLL students living largely in poverty in an ethnically diverse public school system in the U.S. Specifically, we followed students longitudinally from kindergarten through eighth grade, and asked how the number of years it took DLLs to be reclassified in the school system as no longer needing ESOL services is related to eighth grade academic outcomes (GPA and math and reading test scores), and retention (repeating a grade) in middle school (sixth through eighth grade).

### 1.1. DLL Reclassification in School Systems

Much of a DLL student’s experience in the school system depends on their eventual reclassification as no longer needing ESOL services. For studies like the present that used student reclassification as a marker for English proficiency, it is important to understand how DLL students are initially classified. Due to the 1974 Lau v. Nichols Supreme Court ruling and the 2015 Every Student Succeeds Act ruling, individual school systems must take steps to teach English to students not yet fluent in the language of instruction and provide access to the general curriculum (Thompson, 2017). During this time, DLLs receive extra language support while simultaneously learning the broad curriculum taught to all students. When DLLs reach a school’s predetermined English proficiency level, they are reclassified as English proficient and cease receiving ESOL services (Saunders & Marcelletti, 2013). For some students, exiting the ESOL program is not an issue, as their English level is thoroughly developed, but for others, losing the extra language supports that the ESOL program provides can be difficult. There may be a significant gap in performance expectations between the ESOL program goals and state-mandated goals for general classes, and newly reclassified DLL students may struggle to meet these new goals (Abedi & Dietel, 2004; Saunders & Marcelletti, 2013).

In general, the average time it takes for a DLL student to reclassify as English proficient is between four and seven years, with the chance of full reclassification dropping dramatically after nine years (Estrada & Wang, 2017). Students who are unable to reach English proficiency in this time period are at risk of becoming “long-term English learners” (LTELs). LTELs are DLL students who have been enrolled in the U.S. school system for six or more years but who have been unable to reclassify as English proficient (Chen-Gaddini & Burr, 2016). Research has found that LTELs’ English language acquisition often slows over time, and while some of these students reach high levels of oral English proficiency, they cannot successfully reclassify due to insufficient academic language abilities (typically reading) in English (Shin, 2024; Thompson, 2017). In a study looking at LTELs in secondary school, Callahan (2005) found that the students often had significantly lower academic performance than other DLLs and non-DLL students. In addition to the initial academic consequences of DLLs becoming LTELs, LTEL status itself comes with long-term negative effects on graduation rates and academic aspiration (Shin, 2024).

One challenge is a lack of consensus on the exact level that DLL students must reach for reclassification, since the definition of English proficiency depends somewhat on the school system attended and the ESOL programs operating there. Under the federal No Child Left Behind Act and the Every Student Succeeds Act, all local education systems must administer English language proficiency assessments to their DLLs each year before reclassification, and states must define their English-proficient performance standards on this assessment (Thompson, 2017). However, ELP reclassification standards vary by state, district, and school, making it difficult to compare results across studies.

### 1.2. Timing of English Proficiency

Previous research has used longitudinal data to track the relationship between scores on academic assessments and DLLs’ English proficiency (Jiménez-Castellanos et al., 2014; M. Kieffer, 2012; Llosa, 2012; Roberts et al., 2010; Slama, 2012; Spack, 2004). These studies show the importance of using long-term tracking methods to produce a more accurate picture of DLLs’ developing English skills. Tracking DLLs’ language skills over an extended period of time is critical for accurately mapping their academic trajectories and identifying relationships between time to reclassification for DLLs and later academic outcomes. Within the overarching period in which DLLs’ language development is being tracked, it is essential to examine how early English proficiency levels affect students’ educational outcomes in middle school, as this period of schooling is where students experience more advanced coursework and rigorous academic pressure (Mosqueda & Maldonado, 2013). M. Kieffer (2012) used latent growth models and longitudinal data on a cohort of Spanish-speaking DLLs in the U.S. to investigate how early oral language proficiency in Spanish and English predicted later literacy levels, controlling for family SES. Kieffer reported that both Spanish and English proficiency in kindergarten were predictive of English reading levels in third through eighth grade, showing that both L1 and L2 development are crucial for academic achievement.

Overall, the timing with which DLL students acquire English proficiency is predictive of scores, including literacy and test scores in elementary and middle school (Guglielmi, 2008; Mosqueda & Maldonado, 2013; Winsler et al., 2023). Ardasheva et al. (2012) investigated the long-term predictive power of early English proficiency for the middle school academic achievement of DLLs compared to native English speakers. In this study, multilevel analyses were performed on data from 17,470 native-English-speaking students, 558 current DLL students, and 500 former DLL students who had acquired English proficiency by the start of middle school. Results found that former DLLs who reached English proficiency significantly outperformed current DLLs and native English-speaking students in reading (effect sizes: 1.07 and 0.52) and mathematics (effect sizes: 0.86 and 0.42) (Ardasheva et al., 2012). Upon reaching adequate proficiency in the language of schooling and testing, DLLs no longer experience academic disadvantages.

Likewise, Halle et al. (2012) examined DLLs’ longitudinal academic performance through eighth grade. This study reported the early English proficiency levels of a large, nationally representative sample of DLL students starting in kindergarten (*N* = 19,890) and tracked differences in academic scores, particularly in reading and math, throughout middle school. Growth curve analyses found that test scores correlated with the grade at which students acquired English proficiency. DLLs with faster English acquisition speeds, specifically, no later than 1st grade, had higher later academic achievement compared to DLLs who acquired English at a later time period (Halle et al., 2012). Limitations of Halle et al., however, include the narrow measurement/definition of English proficiency available in the ECLS-K (passing a small number of researcher pre-screener items) and the fact that the outcome measures used were decontextualized, research-administered assessments. Here, we build upon this work by using more comprehensive English language assessment batteries and including ecologically valid, real-world achievement measures, metrics used by the students’ own schools when making progress and promotion decisions.

Controlling for individual student factors is critical when tracking the relationship between the timing of English acquisition and academic achievement, as many variables are correlated with both timing of English proficiency and academic outcomes (Kim et al., 2014; Sharkey & Layzer, 2000). In a longitudinal study, Kim et al. (2014) identified several factors predictive of DLLs’ faster English acquisition/ESOL exit, including higher initial L2 proficiency in kindergarten, not being in poverty, not being Hispanic/Latino or Black, stronger cognitive, language, and socio-emotional skills at age 4, and higher maternal education. Furthermore, attending high-quality public school pre-K has also been shown to increase English language acquisition speed for DLLs (Ansari et al., 2017; Miller, 2017; Winsler et al., 1999). Family and neighborhood poverty, in particular, are well known to be one of the, if not the strongest, predictors of student academic achievement in schools (Duncan & Magnuson, 2013; S. F. Reardon, 2018).

### 1.3. Retention

In addition to standard academic outcomes such as test scores and GPA that have been explored in previous studies, the current study includes grade retention as an additional academic outcome. Grade retention has negative effects on downstream student achievement and motivation (Diris, 2017; García-Pérez et al., 2014; Hughes et al., 2018; Kretschmann et al., 2019). DLLs have a higher risk of being retained due to several individual factors, such as low English literacy skills and first-generation immigrant status (Bowman-Perrott et al., 2009; Tillman et al., 2006; Willson & Hughes, 2006). In every grade except for kindergarten, DLLs are overrepresented in the proportion of students retained (13%) relative to their makeup in the overall student population (10%) (U.S. DoED, 2016). Much of this retention can be traced back to lower (English) literacy scores and struggles with passing high-stakes, standardized reading tests sometimes used for promotion decisions (Bowman-Perrott et al., 2009). However, it is important to note that these retention statistics typically do not control for socioeconomic status, a known predictor of retention (Locke & Sparks, 2019; Winsler et al., 2012), and they are calculated cross-sectionally, each grade individually (U.S. DoED, 2016). This means that they include immigrant students in each grade who just recently arrived at their school with very limited English skills. Much less is known about academic retention longitudinally for those DLLs who arrived at school early on in kindergarten, the group of students studied in the current investigation. Some studies with younger DLL students enrolled in U.S. preschool programs have found that DLL students have *lower* kindergarten retention rates compared to their non-DLL peers, perhaps due to the particularly good behavioral skills (as rated by preschool teachers) typically found in DLLs (Winsler et al., 2012). While DLL students are less likely to be retained compared to native English speakers, DLLs with lower language skills (in English or Spanish) at age four are *more* likely to repeat kindergarten than DLLs with stronger language skills, after controlling for children’s actual performance (teacher grades) during their first time in kindergarten (Winsler et al., 2012). The current study examines how the timing of reclassification for DLLs is related to retention through middle school.

### 1.4. The Present Study

The current study is a longitudinal extension (through middle school—eighth grade) of our prior work that examined elementary school outcomes (third–fifth grade) associated with the timing of English acquisition for DLLs in Miami, Florida (Winsler et al., 2023). Winsler et al. (2023) investigated how the grade at which DLLs (*N* = 17,548) were reclassified (i.e., exited ESOL programs) related to later academic outcomes for DLLs in third, fourth, and fifth grade in a predominantly Hispanic/Latine and low-income sample. We found that after controlling for gender, ethnicity, poverty status, and child school readiness skills at age four, DLL students who reached English proficiency earlier had significantly higher fifth-grade performance in math and reading tests, had significantly better GPAs, and were less likely to be retained than DLLs who attained English proficiency later, and that every year/grade mattered (Winsler et al., 2023). Furthermore, moderation effects were found—the timing of English proficiency was more strongly related to fifth-grade outcomes for students *not* in poverty, for Latine (compared to Black) students, and for students with stronger cognitive skills at age four.

The present study longitudinally follows the same sample through eighth grade, controls for similar covariates, and examines similar outcomes to Winsler et al. (2023) to see if the grade at which DLL students reclassify continues to have long-lasting links with later academic achievement at the end of middle school. This is an important extension of prior work for a number of reasons. First, most prior work on the grade at which English proficiency is attained has examined either earlier elementary school or later high school outcomes (Agirdag, 2014; Callahan, 2005; de Jong, 2004; Lindholm-Leary & Hernández, 2011; Tabors et al., 2003; Zaff et al., 2020), with very few studies focusing on the middle school academic achievement of former DLLs. Middle school is a critical time to understand for LTELs who have not yet reached English proficiency (Chen-Gaddini & Burr, 2016; Thompson, 2017). Second, Winsler et al. (2023) had a critical limitation in that they only examined the grade level (e.g., second or third) at which the students exited ESOL, ignoring the temporal information pertaining to students who repeated a grade one or more times (thus having additional years of schooling). The current study converted grade levels into the *number of years* that DLLs spent in ESOL programs as a superior, continuous measure of time. Third, it could be the case that the timing of ESOL exit only matters for DLLs’ academic performance in elementary school and that other predictors of performance come into play for students in eighth grade. Thus, we felt it would be an important contribution to the literature to see if the timing of English mastery *continues* to matter for student outcomes at the end of middle school.

We investigated two main research questions. First, after controlling for poverty, gender, race/ethnicity, and disability status, to what extent does the total number of years that DLL students take to reach English proficiency predict later academic outcomes (grade point average, standardized test scores, and retention) at the end of middle school—eighth grade? Based on the previous literature, we hypothesized that children who reclassify earlier would experience stronger educational outcomes at the end of middle school compared to their DLL peers who reach English proficiency later. Second, is the effect of the timing for English proficiency on DLLs’ eighth-grade outcomes moderated by poverty status, and/or race/ethnicity? As found in Winsler et al. (2023), we expected that race/ethnicity and poverty might again account for significant achievement variance, leaving the timing of students’ exit from ESOL programs to more strongly predict academic outcomes for Latine students (compared to Black students) and students not in poverty (compared to those in poverty).

## 2. Method

### 2.1. Participants

*Background.* This study used data from the Miami School Readiness Project (MSRP), a longitudinal study that has followed five cohorts of children in Miami-Dade County since 2002 (Winsler et al., 2008). The MSRP included 4-year-old children in Miami-Dade County from 2002 to 2007 who were enrolled in public school pre-K programs or who received subsidies to attend childcare in the community and followed these children longitudinally throughout their schooling (Winsler et al., 2008). The data on these children extends to 8th grade, including children who either skipped a grade or were retained. Children were administered school readiness assessments at age four to assess their cognitive, socio-emotional, and motor skills (discussed below). Data and scores assessing English proficiency and academic performance measures were gathered each year from kindergarten onwards from the school system (discussed below) (Winsler et al., 2023).

*Sample.* The current study sample started with every DLL student in the MSRP (*N* = 19,116). For this project focusing on middle school students, students needed to show up in middle school and have data for at least one academic outcome in 8th grade to be included in the study (*N* = 14,852). The school system classified students as DLLs if their parents indicated that they spoke a language other than English predominantly at home, and they had a valid score from their school’s English proficiency assessment in kindergarten (Kim et al., 2014; Winsler et al., 2023). Students also had to have information on when they reached full English proficiency to be included in this sample, as measured by their ESOL exit grade and total years in ESOL (discussed below).

This sample consisted of children who progressed throughout their schooling in a typical manner, children who were retained a grade either once or twice, and children who skipped a grade. Students who had delayed their kindergarten entry were excluded from this sample. This was done because such students would always have fewer years in their ‘Years in ESOL’ variable, which would skew the time to reclassification data. Additionally, retention was an outcome variable, and the data on the student’s first attempt at a grade was used if that child was retained in an outcome year. The sample is evenly divided by gender (47.7% female, 52.3% male). The majority of participants come from low-income backgrounds (84.7%), defined as receiving free- or reduced-price lunch (FRL) in 6th or 8th grade. Finally, DLL participants in this study are 87.4% Hispanic/Latino, 8.4% Black, and 3.2% White/Asian/Other. Students attended about 60 different middle schools in one large, ethnically diverse urban school district (68% Hispanic, 24% Black, 8% White; 78% FRL; 15% receiving ESOL services). ESOL services for DLLs in this district at the time varied somewhat from school to school but did not vary much from year to year. Although some schools provided some support for students’ L1 in the form of two-way immersion programs, most bilingual education services were focused on structured exposure to English. Unfortunately, we did not have data on which children received which types of ESOL services.

### 2.2. Measures

*English Proficiency*. The grade at which DLL students became English proficient was operationally defined in this study as the grade in which they reached ESOL level 5. ESOL levels in Miami-Dade County are Novice (Level 1), Low Intermediate (Level 2), High Intermediate (Level 3), Advanced (Level 4), and Independent (Level 5) (M-DCPS, 2018). Schools assessed DLL students’ ESOL levels each year at the beginning of the year, and these levels are determined by tests designed to assess students’ English language proficiency in four language domains: listening, speaking, reading, and writing. DLL students are assessed annually until they reach Level 5, at which point they exit the ESOL program. We received end-of-year data on ESOL levels, so we interpret the first year that they appeared as a ‘5’ as the year in which they became proficient. An important note is that although the assessments used to evaluate the students’ ESOL level changed from the beginning to the end of the present study, each level’s functional meaning remained constant.

From 2003 to 2007, the Miami-Dade County Oral Language Proficiency Scale-Revised (M-DCOLPS-R) test was used to assess the ESOL level of DLL students and was administered on an individual basis before kindergarten entry (Florida Department of Education [FDOE], 2009). The M-DCOLPS-R contains 25 items, with raw scores ranging from 1 to 25, and for each correct answer, the student receives 1 point. Correct responses on test items showed that the DLL student displayed both understanding and linguistic control of vocabulary, structure, and pronunciation. If students scored four or lower on this assessment, they were placed in ESOL level 1. Likewise, scoring 20 or higher in the assessment corresponded with ESOL level 5, which indicated that the DLL student was sufficiently English proficient and did not require ESOL services upon school entry. In 3rd grade, in those early cohorts/years, DLLs also had to test into the 32nd percentile on both the Reading Comprehension and Language Mechanics Subparts of the Metropolitan Achievement Test to reach ESOL level 5 (Florida Department of Education [FDOE], 2009).

The Comprehensive English Language Learning Assessment (CELLA) began to be administered to DLL students in Miami-Dade County every spring from 2006 to 2015 (AccountabilityWorks, 2015; FDOE, 2015). This assessment was designed to support program accountability and provide data for tracking the progress of DLLs over time. This assessment also supplies the information that determines if a DLL student is eligible to exit the ESOL program (FDOE, 2015). The CELLA has four separate levels differentiated for grades K-2, grades 3–5, grades 6–8, and grades 9–10, which also consist of a set of subtests used to assess listening, speaking, reading, and writing (AccountabilityWorks, 2015; FDOE, 2015). Both the M-DCOLPS-R and the CELLA place DLL students at an ESOL level ranging from 1 to 5. We used the grade in which DLL students were classified as ESOL level 5 as a measure of when they attained English proficiency, and this was treated continuously in analyses.

*Timing of English Proficiency.* Two variables were used to investigate when DLLs obtained English proficiency. First, a descriptive variable was created that showed the *grade* in which each DLL student reached ESOL level 5 and exited the ESOL program, coded as 0 = kindergarten, 1 = first grade, 2 = second grade, 3 = third grade, 4 = fourth grade, 5 = fifth grade, 6 = sixth grade, 7 = seventh grade, 8 = eighth grade and beyond. This variable did not differentiate students who were retained in a grade at any point. For example, a DLL who reached English proficiency and exited the ESOL program during their second time in 1st grade was still coded as a 1 (as was done in Winsler et al., 2023). Second, a superior continuous variable was created to show the total *number of years* a DLL student spent in the ESOL program, coded as 1 = one year, 2 = two years, 3 = three years, and so on. This variable did account for students who were retained at some point. For example, if a DLL reached full English proficiency and exited the ESOL program during their second time in 1st grade, they would be coded as a 3 (kindergarten + 1st grade + repeated 1st grade). Students who were missing from the school system for a year or two but returned with an ESOL level still below 5 were kept in the sample, but students who were missing for several years and returned already at a level 5 were excluded. This was done because we were unable to know how many of those missing years the student was in ESOL and how many were not. This variable can be seen as time-sensitive and continuous, and this is what was used for inferential statistical analyses.

### 2.3. Academic Outcomes

*Grade point average.* The schools provided data on students’ teacher-assigned grades across all subjects for each year, starting from kindergarten. In every grade after kindergarten, students were given an end-of-year GPA that was determined by the average of their grades in all subjects, including, for example, math, science, art, music, social studies, physical education, reading, writing, and language arts. We used the student’s 8^th^-grade GPA as an outcome variable, calculated on a scale of 1 (‘F’) to 5 (‘A’). As GPA is an average of school performance across many different subject areas, it provides an overall continuous indicator of academic performance in coursework and in the classroom. Consistent with Dickinson and Adelson (2016), despite the additional noise introduced from variance across teachers and different standards used to assign grades, we see GPA as a valid measure of an important component of overall academic achievement in context. This measure provides unique information about student performance above and beyond what is measured by standardized tests, and is commonly used successfully as a predictor of later student performance.

*Standardized tests.* Students in this state took the Florida Comprehensive Achievement Test (FCAT; Human Resources Research Organization & Harcourt Assessment, 2006). The FCAT has an internal consistency using Cronbach’s Alpha of 0.91 for reading and 0.88 for math. This is a mandatory assessment for all students to take in Florida at the end of 6th, 7th, and 8th grade, and scores include a reading and math scale ranging from 100 to 500 for each scale. During the 2010–2011 school year, students started to receive a newer version of the FCAT, the FCAT 2.0. Then again, in the 2015–2016 school year, students were administered a different test, the Florida Standards Assessment (FSA), based on the new Florida standards. The DLL students were administered one of these three tests during middle school, depending on their grade and cohort. Because the standard score scale and standardization differed notably across the three versions, we used the ordinal 1–5 scale, which was consistently scaled and used by the school system to be the same across all versions/years. On this scale, 1 and 2 indicate the student is not performing at grade-level expectations, 3 means the student is performing at grade level, and 4 and 5 mean above-grade-level expectations are met.

*Middle School Retention.* In addition to GPA, schools also provided de-identified records on which grade level students were placed in each year. A student was deemed to have been retained if they repeated a grade (Winsler et al., 2012), and an indicator in the data was created. For example, a student who advanced on time from 6th to 7th grade would have end-of-year grades in 6th grade, appear in 7th grade the next year, and then have grades at the end of 7th grade. On the other hand, a student retained in 6th grade would have end-of-year grades in 6th grade but then appear in the same grade level again the next academic year and have end-of-year grades for 6th grade a second time. We looked at whether a student was retained in each individual grade for descriptive purposes and then designated a variable for ever being retained in middle school from 6th to 8th grade as an overall outcome (0 = no, 1 = yes).

### 2.4. Covariates

*Demographic variables.* Demographic variables known to be related to both the speed of English language acquisition and academic achievement, such as poverty (FRL) status, gender, ethnicity, and disability status, were added as covariates. Free/reduced-price lunch status was used to operationalize poverty status (i.e., receiving FRL in 6th (1 = yes, 82.9%) indicated that the participant was from a lower-income background than those who did not receive FRL (0 = no, 17.1%). School records provided information on the gender and ethnicity of participants, and as such, ethnicity had three categories: Hispanic/Latino, African American/Black, and White/Asian/mixed/other. Student disability status was also included, which encompasses learning and language disabilities but does not include gifted status. The student’s 6th-grade disability status was used as an overall indicator, with students either being categorized as having a disability or not (1 = yes, 16%; 0 = no, 81.9%). In our prior work (Winsler et al., 2023), preschool school readiness assessments were included as covariates because they were still significantly related to fifth-grade outcomes. They were not included here because they did not contribute significant variance in predicting eighth-grade outcomes, and we wanted to avoid their missing data to maximize the sample.

## 3. Results

### 3.1. Descriptive Analysis

First, we descriptively report the percentage of the DLL sample that reached the threshold and were considered English proficient, as well as their total number of years in the ESOL program. All descriptive analyses were run in SPSS 29. Table 1 shows the grade level at which the DLLs exited ESOL services and the number of years in ESOL, which accounts for students being retained a grade. As seen in the table, 29% of students reached proficiency in kindergarten, and another 24% reached proficiency in first grade. A further 38% of students reached English proficiency before the end of elementary school, while the remaining 9% of DLLs reached proficiency during middle school. Similarly, 29% of students spent only one year in the ESOL program, and 23% spent two years in the program before exiting. Of the DLLs who were in the ESOL program for more than two years, 37% exited the program within five years, while only 10% of DLLs were in the program for six or more years.

### 3.2. Multivariate Analyses

Mplus was used for multiple regression analyses (and logistic regression analysis for the dichotomous outcome of retention) to utilize full information maximum likelihood (FIML) to handle any missing data on predictors. Mplus’ Type = COMPLEX function also allowed for us to account for school-level clustering, with students being nested in different middle schools. The year that the DLL acquired English proficiency, analyzed linearly, was the main predictor of interest. We also analyzed years using a quadratic function (years in ESOL squared) to test for potential nonlinear effects (Winsler et al., 2023), but nonlinear effects were never significant, so we removed that predictor from the models. Finally, in a second batch of the regression models, we added interaction terms, one at a time, to see if the effect of years in ESOL on eighth-grade outcomes was similar for (a) those in poverty vs. not, and (b) Black vs. Latino DLLs (White/other students were excluded due to very small cell sizes).

**Figure 1:**
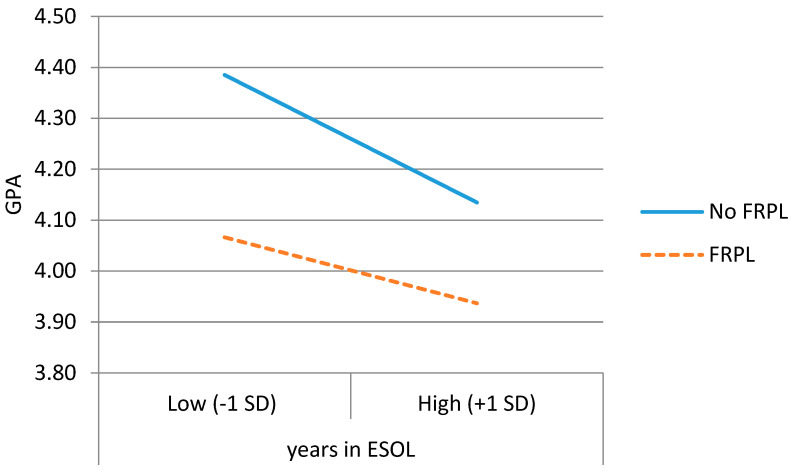
DLLs’ 8th-grade GPA by total years in ESOL for those in poverty (FRPL) or not.

**GPA.** The first column of Table 2 shows the regression results for the effect of total years in ESOL on DLLs’ eighth-grade GPAs, including covariates. Years in ESOL, gender, ethnicity, poverty status, and disability status were significant predictors of eighth-grade GPA. Compared to Hispanic/Latino DLL students, White DLL students’ eighth-grade GPA was 0.18 points higher, and Black DLL students’ GPA was 0.17 points lower. DLL girls’ eighth-grade GPA was 0.25 points higher than that of DLL boys, and DLL students with disabilities had an eighth-grade GPA that was 0.13 points lower than that of DLL students without disabilities. Additionally, DLL students in poverty had an eighth-grade GPA that was 0.28 points lower than that of DLL students who were not. Importantly, net of all covariates, DLL students’ eighth-grade GPA decreased by 0.033 points for each additional year that English proficiency was not reached. The interaction between ethnicity and total years in ESOL was not significant, but the interaction between poverty and total years in ESOL was. The timing of English proficiency was more strongly related to eighth-grade GPA (steeper slopes) for DLLs who were not in poverty. Figure 1 depicts the interaction effect for GPA.

**Standardized Reading Test Scores.** Table 2 also shows the regression results for the effect of total years in ESOL on DLLs’ eighth-grade reading scores. The time to proficiency, gender, ethnicity, poverty status, and disability status were significant predictors of eighth-grade reading scores. Compared to Hispanic/Latino DLL students, White DLL students’ eighth-grade reading scores were 0.22 points higher, and Black DLL students’ scores were 0.42 points lower. DLL girls’ eighth-grade reading scores were 0.15 points higher than those of DLL boys, and DLL students with disabilities had eighth-grade reading scores that were 0.85 points lower than those of DLL students without disabilities. Additionally, DLL students in poverty had an eighth-grade GPA that was 0.42 points lower than that of DLL students not in poverty. Of interest, after controlling for the above covariates, DLL students’ eighth-grade reading scores (measured on a scale of 1–5) decreased by 0.14 points for each additional year that English proficiency was not reached. The interaction between ethnicity and the timing of proficiency was not significant, but the interaction between poverty and total years in ESOL was. Similar to the pattern for GPA depicted in Figure 1, the timing of English proficiency was more related to reading scores (steeper slopes) for DLLs who were not in poverty.

**Standardized Math Test Scores.** Table 2 shows the regression results for the effect of ESOL exit grade on DLLs’ eighth-grade math scores. The effects of covariates (gender, ethnicity, poverty status, and disability status) on math were similar to those above for reading. Importantly, DLL students’ eighth-grade math (also measured on a 1–5 scale) decreased by 0.07 points for each additional year that English proficiency was not reached. The interaction between ethnicity and total years in ESOL was not significant, but the interaction between poverty and total years in ESOL was. The timing of English proficiency was more strongly related to later math scores (steeper slopes) for DLLs who were not in poverty.

**Middle School Retention.** Table 3 shows the logistic regression results for predicting being retained in middle school. There was a significant association between the timing of English proficiency and being retained in sixth, seventh, or eighth grade. Total years in ESOL and gender were significant predictors of retention in middle school. For each additional year that DLLs did not reach the English proficiency threshold, there was an almost 11% increase in the odds of retention in middle school. DLL boys were three times more likely to be retained in middle school than girls. The interaction between ethnicity and the timing of proficiency was not significant, but the interaction between poverty and years in ESOL was. The timing of English proficiency was more strongly related to retention for DLLs *not* in poverty compared to DLLs in poverty. Each additional year that DLLs not in poverty were delayed in acquiring proficiency was associated with 60% greater odds of middle school retention (while DLLs in poverty did not experience significantly greater odds of retention with additional years in ESOL).

## 4. Discussion

Dual Language Learners who speak a language other than English at home in the U.S. have the task of mastering the instructional language of English in school. When they reach the threshold for mastery determined by the school system, DLLs are reclassified. This study examined how the timing for DLL students to acquire English proficiency is related to later eighth-grade academic performance. We used several measures of students’ academic achievement and focused on a diverse sample of DLLs while controlling for known factors that predict achievement and the speed of English language acquisition, such as gender, race/ethnicity, poverty status, and disability status. The overarching finding from this study was that earlier English acquisition for DLLs is better for eighth-grade academic performance, and this supports prior research showing earlier L2 acquisition positively influences academic performance for DLLs (Halle et al., 2012; O’Connor et al., 2018). Comparisons of DLL students’ eighth-grade GPAs, reading scores, and math scores by the year in which they reached English proficiency indicated that DLLs who acquire English in an earlier grade had better middle school performance.

Some studies find that DLL students with lower initial English proficiency catch up to their non-DLL peers and perform similarly later on (M. Kieffer, 2008; Lesaux et al., 2007; S. F. Reardon & Galindo, 2007). The current study, rather than comparing DLLs to non-DLLs, only examined DLLs upon entry to school in kindergarten and, in a way, compared DLLs who exited ESOL services at different times to each other. Those who acquired proficiency earlier in elementary school performed the best on average in eighth grade.

In prior research looking at a similar effect in elementary school, results showed that the English acquisition grade was more predictive of later reading scores than math scores (Halle et al., 2012). The same pattern was observed here, with the coefficients for eighth-grade reading being about twice as large as those for math (although both were statistically significant). There seems to be a general linear decrease in reading achievement for every year that DLL students do not reach English proficiency. For math scores, however, the decrease is smaller, and there seems to be a plateau where students who become proficient in third through fifth grades all perform similarly on eighth-grade math scores.

The current study showed a much larger drop in eighth-grade achievement for students who were not proficient until after sixth grade compared to students who reached proficiency in elementary school. This sharp difference in middle school academic performance between DLLs who reach English proficiency in elementary school and DLLs who do not reach proficiency until middle school may be due to the transition, more rigorous work in middle school, and the academic challenges that LTELs face. For many students, including LTELs, middle school is one of the first instances where students experience more advanced coursework and rigorous academic pressure (Mosqueda & Maldonado, 2013). Previous research has identified the time spent in classes as an important factor in why reaching English proficiency before sixth grade impacts DLLs’ middle school performance. Being English proficient before entering middle school, and thus not being in ESOL classes in middle school, increases the amount of time for more challenging coursework in English language arts and math from sixth grade to eighth grade (Onda & Seyler, 2020). English proficient students can spend more time learning the skills needed to achieve higher scores than DLLs who are not yet proficient, who spend a greater amount of time in ESOL classes. This difference in exposure to challenging coursework may play an essential role in explaining the sharp contrast in eighth-grade performance between before-sixth-grade-proficiency DLLs and after-sixth-grade-proficiency DLLs.

Prior research indicates a range of contributing factors to DLLs becoming LTELs, including insufficient language support programs, unreasonable exit policies, limited opportunities to learn, and limited support for learning/language disabilities (Shin, 2024; Thompson, 2017). For instance, M. J. Kieffer and Parker (2016) found using discrete time survival analyses that DLLs with lower English proficiency or with specific learning disabilities or language impairments took more time to be reclassified, and their probability of reclassification ever was lower. This could be one factor at play in this current study. For this sample, the percentage of students with disabilities is indeed higher for later-proficiency DLLs than for early-proficiency DLLs. For the early-proficiency DLLs, only 11–22% of the students had a disability, while 23–40% of the DLLs not proficient until middle school had a disability. This may shed some light on the factors that lead to DLLs becoming LTELs; however, more research needs to be performed on identifying and supporting these students earlier. An additional factor to be considered for LTELs is that reclassifying decisions may not always be due to their actual English proficiency abilities but due to other bureaucratic processes (Shin, 2024; Thompson, 2017).

In addition to academic performance, retention in middle school was found to be affected by the grade in which DLLs left ESOL in the current study. Prior work suggests that grade retention has a negative influence on students’ academic performance (Hughes et al., 2018; Kretschmann et al., 2019), and DLLs are overrepresented in the population of retained students (Bowman-Perrott et al., 2009; Diris, 2017; García-Pérez et al., 2014; Tillman et al., 2006). English acquisition year/grade, gender, and disability status were each predictors of retention in middle school. Previous studies have shown that individual factors such as poverty status and race/ethnicity were predictive of retention in elementary school (Tavassolie & Winsler, 2019; Winsler et al., 2012); however, these factors were not significant predictors for middle school retention in this study. Perhaps by middle school, initial differences seen by race/ethnicity and SES have become less prominent for predicting retention, and the speed at which these students acquire English makes a major impact on the odds that DLL students are retained in middle school.

As was found in our earlier work that only following students through elementary school (Winsler et al., 2023), poverty moderated the effects of English acquisition timing on eighth-grade academic outcomes. The timing of English learning was related to eighth-grade outcomes for all students, but, as shown in Figure 1, the association was weaker for students living in poverty. This is likely due to the fact that poverty is well known to account for a lot of the variance observed in student academic achievement (S. F. Reardon, 2018), and poverty interacts with other factors to predict academic outcomes for DLLs (Sharkey & Layzer, 2000). For students who do not have the significant risk factor of poverty, ESOL exit grade explains more of the variance in eighth-grade academic outcomes than for students whose performance is already heavily impacted by poverty.

**Limitations.** This present study has multiple strengths, including a large sample of DLLs, high-stakes academic performance outcomes, and grade retention as an outcome of interest. Although this study used an ecologically valid measure of English proficiency during every grade, one variable of interest that could not be included was DLL students’ home language skills and the support of that home language in classrooms. DLLs’ L1 proficiency is generally highly correlated with their L2 proficiency (August & Shanahan, 2006; Winsler et al., 2014), and L1 support in classrooms is seen to be predictive of how quickly DLLs acquire L2 proficiency (Lee & Macaro, 2013). A measure of DLLs’ L1 proficiency would have been a valuable variable to include in this study. Additionally, there was the limitation of not having child-level information about the specific types of language services and bilingual programs in which DLLs participated.

Another limitation of this study that should be acknowledged is that the only DLL students included in this sample were students enrolled in public school pre-k programs at four years old or who received childcare subsidies in Miami-Dade County. DLL students who arrived in the school system at any later date could not be included in the sample due to the longitudinal nature of the student tracking. Additionally, students who moved away from Miami-Dade County could not be included due to the lack of outcome data for them. This made it impossible to account for a certain group of DLLs who entered the school system and ESOL during elementary school, even though their rate of English acquisition would have been beneficial to include in the analyses. However, while other studies do include later-arriving DLLs, not including these students in this sample allowed this study to focus on the long-term effect of English acquisition over time for those entering school in kindergarten as ELLs. Focusing on a sample of students who all entered the school system in kindergarten at the same time allowed us to control for that variable, and while there are benefits to including late arrivers, their scores would have skewed the outcome means in this study.

**Future Avenues for Research.** As earlier English acquisition was significantly predictive of DLLs’ eighth-grade outcomes and L1 support correlates with faster English acquisition speed (Lee & Macaro, 2013; Steele et al., 2017), future research should explore the relationship between home language and middle school achievement. It would be beneficial to understand further how these early language support systems specifically affect middle school and high school outcomes. This is true for students who enter the school system in kindergarten and begin the English acquisition process early, and for students who enter the school system later and may have higher L1 competencies but relatively low or no English proficiency.

Additionally, future research should explore what factors are related to DLLs becoming LTELs and how this affects their academic progress. Specifically, what factors influence students who enter the school system in kindergarten but fail to reach English proficiency by the end of elementary school, and what does this mean for their middle school and high school achievement? LTELs are overrepresented in grade retention and dropout rates (Hanover Research, 2017; Onda & Seyler, 2020), so understanding what specific interventions or supports may be needed is critical to assisting these students and tracking their progress. Furthermore, understanding why LTELs have higher retention rates in secondary education may inform ESOL intervention in elementary school.

Finally, future work should explore how school-level factors and interschool tracking affect DLL students’ English acquisition. Research has shown that the quality of education available to students has become increasingly dependent on the social and economic demographics of the schools that the children attend (Ruiz et al., 2018), and this is no different for DLLs. A profitable future research topic would be to investigate differences in LTELs’ academic performance related to the format and structure of specific ESOL programs experienced by the students. Research examining the achievement of DLLs through middle school does find that ESOL program type, such as English immersion or dual immersion, finds differences based on program type (Valentino & Reardon, 2015). Further, some find that access to programs that include the home language, such as two-way immersion, is beneficial to DLLs’ English acquisition (Serafini et al., 2022), and schools’ ability and willingness to provide these programs are based on many individual factors. Additionally, accurate tracking of DLLs between schools and how these schools differ in language support systems may inform why some DLL students reclassify as English proficient faster than others when switching schools.

In conclusion, the present study suggests that earlier English acquisition, specifically in elementary school, is an important factor in DLLs’ middle school academic success. It should be a goal for educators and policymakers to understand the factors that lead to DLLs acquiring English early and limiting the chance that DLLs become LTELs, including L1 support in schools, tracking school-level characteristics, and access to language interventions where possible. The population of DLLs in the US school system will only continue to grow, so mapping out the factors that lead to success for these students is critical to ensuring their access to equitable education.

## Figures and Tables

**Table 1:** DLL Students who acquired English proficiency in each grade and the total number of years in ESOL.

Grade of ESOL Exit	*n*	% of Total	Total # of Years in ESOL	*n*	% of Total
K	4358	29.3%	1	4296	28.9%
G1	3590	24.2%	2	3410	23%
G2	2724	18.3%	3	2600	17.5%
G3	2265	15.3%	4	2441	16.4%
G4	358	2.4%	5	490	3.3%
G5	338	2.3%	6	289	1.9%
G6	558	3.8%	7	382	2.2%
G7	592	4.0%	8	613	4.1%
G8	69	0.5%	9	301	2%
-	-	-	10	30	0.2%
Total	14,852	100%	Total	14,852	100%

**Table 2:** Regression results predicting 8th-grade GPA, reading scores, and math scores by total years in ESOL.

	GPA		Reading		Math	
Predictors	*B*	*SE (B)*	*B*	*SE (B)*	*B*	*SE (B)*
Intercept	4.38	0.028	3.92	0.053	3.06	0.073
Male	−0.253 **	0.014	−0.154 **	0.021	0.061 *	0.024
White vs. Latino	0.184 **	0.032	0.224 **	0.055	0.291 **	0.075
Black vs. Latino	−0.171 **	0.047	−0.422 **	0.051	−0.254 **	0.062
Poverty	−0.278 **	0.022	−0.415 **	0.039	−0.377 **	0.048
Disability	−0.125 **	0.024	−0.848 **	0.038	−0.606 **	0.037
**Years in ESOL**	−0.033 **	**0.004**	−0.141 **	**0.006**	−0.072 **	**0.008**
Years in ESOL × Black/Latino ^a^	0.002	0.008	0.008	0.015	−0.016	0.017
**YearsESOL** **× Poverty ^a^**	0.029 **	**0.008**	0.039 *	**0.015**	0.050 *	**0.022**

** *p* < 0.001 * *p* < 0.05. Note. ESOL = English for Speakers of Other Languages. Latino served as the reference group. ^a^ An interaction term was included in separate models for interactions; White/other students were excluded from the interaction models due to insufficient numbers.

**Table 3:** Logistic regression predicting retention in 6th, 7th, or 8th grade.

Variable	Odds Ratio	SE
Male	3.082 **	0.219
White vs. Latino	0.328	1.02
Black vs. Latino	1.022	0.377
Poverty	2.111	0.381
Disability	1.147	0.295
**Total Years in ESOL**	1.118 *	**0.052**
Years in ESOL × Black/Latino ^a^	1.101	0.096
**Years in ESOL × Poverty ^a^**	0.705 *	**0.141**

** *p* < 0.001 * *p* < 0.05. Note. ESOL = English for Speakers of Other Languages. Latino served as the reference group. ^a^ An interaction term was included in separate models for interactions; White/other students were excluded from the interaction model due to insufficient numbers.

## Data Availability

Data from this study are not available due to their proprietary nature, and restrictions on our data-sharing agreement with the public school system involved. Inquiries can be sent to the first author.

## Abbreviations

The following abbreviations are used in this manuscript:

DLL	Dual Language Learners
ESOL	English for Speakers of Other Languages
L1	First Language
L2	Second Language
LTEL	Long-Term English Learner
GPA	Grade Point Average
FRL	Free or Reduced Price Lunch
CELLA	Comprehensive English Language Learning Assessment
MDCPS	Miami-Dade County Public Schools
EL	English Learner
M-DCOLPS-R	Miami-Dade County Oral Language Proficiency Scale-Revised
